# Network structure of mobile phone addiction and anxiety symptoms among rural Chinese adolescents

**DOI:** 10.1186/s12888-023-04971-x

**Published:** 2023-07-10

**Authors:** Jingjing Wang, Yunjiao Luo, Na Yan, Yuhao Wang, Blen Dereje Shiferaw, Jie Tang, Yifei Pei, Qian Chen, Yiyang Zhu, Wei Wang

**Affiliations:** 1 School of Public Health, Xuzhou Medical University, 209 Tong Shan Road, 221004 Xuzhou, Jiangsu China; 2 Key Laboratory of Human Genetics and Environmental Medicine, Xuzhou Medical University, Xuzhou, China; 3 Engineering Research Innovation Center of Biological Data Mining and Healthcare Transformation, XuZhou Medical University, 221004 XuZhou, Jiangsu China

**Keywords:** Mobile phone addiction, Anxiety, Sleep quality, Adolescents, Network analysis

## Abstract

**Background:**

The incidence of mobile phone addiction among adolescents in rural areas of China is increasing year by year, and has already exceeded that of some cities. And phone addiction increases the risk of anxiety and poor sleep. Therefore, this study used network analysis to investigate the relationship between mobile phone addiction and anxiety symptoms, and the predictability to sleep quality.

**Methods:**

From September 2021 to March 2022, a total of 1920 rural adolescents in Xuzhou, China were included. The survey included information on phone addiction, anxiety symptoms, and sleep quality. Network analysis was used to estimate the network structure of adolescents’ mobile phone addiction and anxiety symptoms. LOWESS curve and linear regression were used to test the predictive ability of node-centrality on sleep quality.

**Results:**

In the network of mobile phone addiction-anxiety symptoms, the most influential symptoms were Failure to cut down the time, Anxiety if not used for some time, and Alleviate loneliness. Irritability was the most prominent bridging symptom. Gender difference had no effect on network structure. Nodes in the network are not predictive of sleep quality.

**Conclusion:**

Failure to cut down the time is the most important symptom, suggesting that measures should be taken to reduce the amount of time spent on mobile phones. For example, increase outdoor exercise, increase the real company of friends and family, in order to reduce the occurrence of mobile phone addiction and anxiety.

**Supplementary Information:**

The online version contains supplementary material available at 10.1186/s12888-023-04971-x.

## Introduction

In recent decades, the use of mobile phone has brought great convenience to people’s life, and the number of mobile phone user are constantly increasing. As of August 2022, the size of China’s mobile Internet users has reached 10.47 billion People, where children and adolescents aged 10 ~ 19 account for 13.5% of the total Internet population [1]. Teenagers are one of main groups of mobile phone users, which are also the most vulnerable victims to the mobile phone addiction [2]. According to relevant surveys, the prevalence of problematic smartphone use among adolescents rose from 6.3% [3] in 2011 to 16% [4] in 2016, which suggests mobile phone addiction among adolescents need urgent attention.

Mobile phone addiction is considered to be an important cause of various psychological and behavioral adjustment problems, especially in adolescents [5]. In recent years, there have been an increasing number of studies on mobile phone addiction among rural adolescents [6, 7]. Interestingly, one representative study found that although there are fewer Internet resources in rural areas, the rate of Internet addiction among rural teenagers is significantly higher than that in urban areas [8]. Several studies have also confirmed that rural areas are a significant predictor of mobile phone addiction [9–11]. However, some problems caused by mobile phone addiction, such as poor academic performance and irrational procrastination, will lead to great academic distress for adolescents [12]. Also, excessive use of mobile phones is more likely to cause headaches, anxiety, lack of concentration and poor sleep quality [13].

Among the many problems caused by mobile phone addiction, the emergence of anxiety symptoms is particularly noteworthy. A cross-sectional study in China found that the prevalence of anxiety among adolescents increased significantly from 2016 to 2020 [14], which brought about the interference of sleep, relaxation and enjoyment of life [15]. It is worth noting that there seem to be a bidirectional relationship between mobile phone addiction and anxiety symptoms [16]. Since mobile phones can alleviate negative emotions, anxious individuals become increasingly dependent on them, eventually leading to addiction [17, 18]. In turn, excessive mobile phone dependence makes addicts feel scared and anxious whenever they are away from their phones [16, 19]. The cognitive-behavioral model argues that an individual’s cognition and emotions could be affected by behavioral problems [20], which indicated those who have mobile phone addiction are more likely to experience high levels of tension and anxiety [21]. Gender differences in psychological distress have also been the focus of relevant research [22]. On the one hand, due to biological differences between men and women, such as hormones and cortisol levels, this may be reflected in emotions and behavior [23]. Women may suffer greater sadness and anxiety than males do, however, since they are more sensitive to stress and pain [24]. Therefore, there is also a need for research on gender differences.

At present, the investigation of mobile phone addiction and anxiety symptoms of rural adolescents is obviously insufficient. Previous studies mostly discussed mobile phone addiction and anxiety as two independent individuals [25], without adequately analyzing their internal mechanisms and relationships. However, we wish to focus on the network structure (i.e., the web of relationships between symptoms) and its effect on the network state (i.e., the activation of symptoms). The network analysis approach fits our needs exactly, allowing us to consider mental health as an emergent property of the interaction of multiple symptoms [26, 27], and the symptom network composed of the various symptoms of the disorder provides rich visual information that allows us to understand the co-morbidity between different mental disorders [28]. Therefore, this study was the first to use network analysis to explore the association between mobile phone addiction and anxiety symptoms, determine that intervention can alleviate the most central (most influential) symptoms of anxiety and mobile phone addiction in rural adolescents, and investigate the relationship between central symptoms and sleep quality.

## Methods and materials

### Participants

From September 2021 to March 2022, four rural middle schools in Xuzhou City, Jiangsu Province, China, were randomly selected for this study. By stratifying different grades, three classes were selected by random sampling for each grade in each school. The selected class as a whole conducted a questionnaire survey on all students. A total of 2004 questionnaires were distributed and 1920 valid questionnaires were returned, with a return rate of 95.81%.

### Measures

Basic demographic data of the subjects, such as gender and age, were collected. The mobile phone addiction of the subjects was investigated by the Chinese version of the Mobile Phone Addiction Index scale (MPAI) [29]. The scale was developed by Louis Leung of the Chinese University of Hong Kong and consisted of 17 items on a 5-point Likert scale, with each item scored from 1 (never) to 5 (always). With higher scores indicating a higher propensity to be addicted to mobile phone. The Cronbach’s α value of the scale in this study was 0.921, which has good reliability.

The Chinese version of the seven-item Generalized Anxiety Inventory (GAD-7) [30] was used to measure the subjects’ anxiety symptoms. The scale has seven entries, each with four options, and scores ranging from 0 (not at all) to 3 (almost every day). The higher the score, the more anxious the subjects were. The Cronbach’s α value of the scale was 0.921.

Sleep quality was measured using the Chinese version Pittsburgh Sleep Quality Index scale (PSQI) [31]. The scale consists of 19 self-rated items and 5 other rated items, where the 19th self-rated item and the 5th other rated item do not participate in the scoring. 18 items make up 7 components, each component is scored on a scale of 0 to 3, and the cumulative score of each component is the total PSQI score, which ranges from 0 to 21. The higher the score, the worse the sleep quality.

### Data analysis

#### Network estimation

All analysis procedures in this study were done using R-studio software. First, the paired Spearman correlation was used to evaluate the association between mobile phone addiction and anxiety symptoms, and the network model was completed using the enhanced least absolute shrinkage and selection operator (eLASSO) approach [32]. The approach employs penalty parameters to achieve sparsity and the extended Bayesian information criterion (EBIC) (i.e., a measure of fit) to determine the optimal set of nearby factors for each node (symptom) [33]. This process requires the use of the R packages “qgraph” [34] and “bootnet” [33]. A visualized network is finally obtained. This network consists of “nodes” and “edges”, where each symptom is considered as a node and the correlation between two symptoms is considered as an edge [35]. The network is automatically built after each node is connected to several other nodes, and it shows the strength of the direct relationship between the nodes [36]. In the network graph, nodes that are more frequently and strongly associated with other nodes are located at the center of the network [37]. Edges of different colors represent different directions of association (red edges represent negative correlations and green edges represent positive correlations) [34]. The strength of the association between nodes is indicated by the edge thickness, where a thicker edge indicates a stronger association between two nodes [38].

There are three main centrality indices to explore the importance of a symptom in the network: strength, closeness, and betweenness [39]. Based on previous studies [37, 40], we chose one of these indices as the centrality index: strength. Strength is the sum of the weights of the edges that connect a node directly to other nodes. This analysis was performed using the “Plot” function in the “qgraph” package of R software, and the results are reported as normalized values (z-cores) [34]. In addition, the bridge centrality of each node in the network was analyzed using the R package “networktools”: bridge strength, a metric that can be used to assess the role a symptom plays in connecting two mental health problems [41]. We also measured the extent to which a given node can be predicted by all its neighbors with the “mgm” R package [42].

#### Estimation of network accuracy and stability

The robustness of the results was confirmed using the case-drop bootstrap procedure in the R package “bootnet”, which continuously removes cases from the original sample while recalculating the centrality index of the nodes in the network. If the centrality index of the nodes does not change significantly after a large percentage of samples are removed, we consider the network structure stable and reliable. The maximum proportion of cases that can be removed from the sample is represented by the CS-C value. In general, the CS-C value shouldn’t be less than 0.25 and should be as high as possible, preferably above 0.5 [33]. In addition, bootstrap differential tests are utilized to examine network property differences.

#### Network comparisons

We used the Network Comparison Test (NCT) to compare the network structure of students of different genders to investigate whether the network structure of mobile phone addiction and anxiety symptoms changed according to gender [43]. The “NetworkComparisonTest” R package was used to do this analysis [44]. First, we used this method to compare the distribution of the absolute values of all edge weights between the two networks to measure whether the overall strength of the networks differed, and then compared the difference in the strength of each edge between the male and female networks [39, 45]. This procedure used the Bonferroni correction.

#### Predicting the value of nodes

We conducted a Spearman correlation analysis for each symptom node (including 17 symptoms of mobile phone addiction and 7 anxiety symptoms) with sleep quality to determine the association between each node and sleep quality. The correlation coefficients obtained from the calculations were considered as the predictive value of these nodes for sleep quality. A higher absolute value of the correlation coefficient indicated that the node had a higher predictive value for sleep quality [46]. To test whether symptoms with high strength can better predict sleep quality, we fit a smoothing curve to the predicted values and node centrality using a locally weighted scatter plot smoother (LOWESS) [47]. And based on the fitting results, linear regression analysis was used to demonstrate whether nodes with high centrality have greater predictive value. It may be confirmed that sleep quality is connected to network integration of anxiety symptoms and mobile phone addiction if the centrality value has a substantial association with the expected value [46].

## Results

Of the 1920 adolescents included in the network, the mean age was 16.28 (Standard Deviation (SD) = 1.70)) years; 837 (43.59%) of them were male; the mean GAD total score was 4.40 (SD = 4.90) and a mean MPA total score was 38.34 (SD = 15.21). The demographic characteristics of the participants are shown in Table 1. The scores for each item on mobile phone addiction and anxiety symptoms are listed in Supplementary Table 1.

**Table 1:** Participant characteristics

Variables	Mean/N	SD/%
Age	16.28	1.70
Male gender	837	43.59
Only child	204	10.63
Learning Stage		
Middle School	826	43.02
High School	1094	56.98
Monthly per capita family income		
≤ 999 yuan	60	3.12
1000–1999 yuan	137	7.13
2000–3999 yuan	378	19.69
4000–5999 yuan	547	28.49
6000–7999 yuan	382	19.90
≥ 8000 yuan	416	21.67
Father’s education		
Primary school and below	130	6.77
Middle school	914	47.60
High school and Specialized secondary school	641	33.39
University and junior college	223	11.61
Master’s degree and above	12	0.63
Mother’s education		
Primary school and below	270	14.06
Middle school	986	51.36
High school and Specialized secondary school	471	24.53
University and junior college	178	9.27
Master’s degree and above	15	0.78
PSQI total	6.29	2.58
GAD total	4.40	4.90
MPA total	38.34	15.21

### Network estimation and strength centrality

**Fig. 1:**
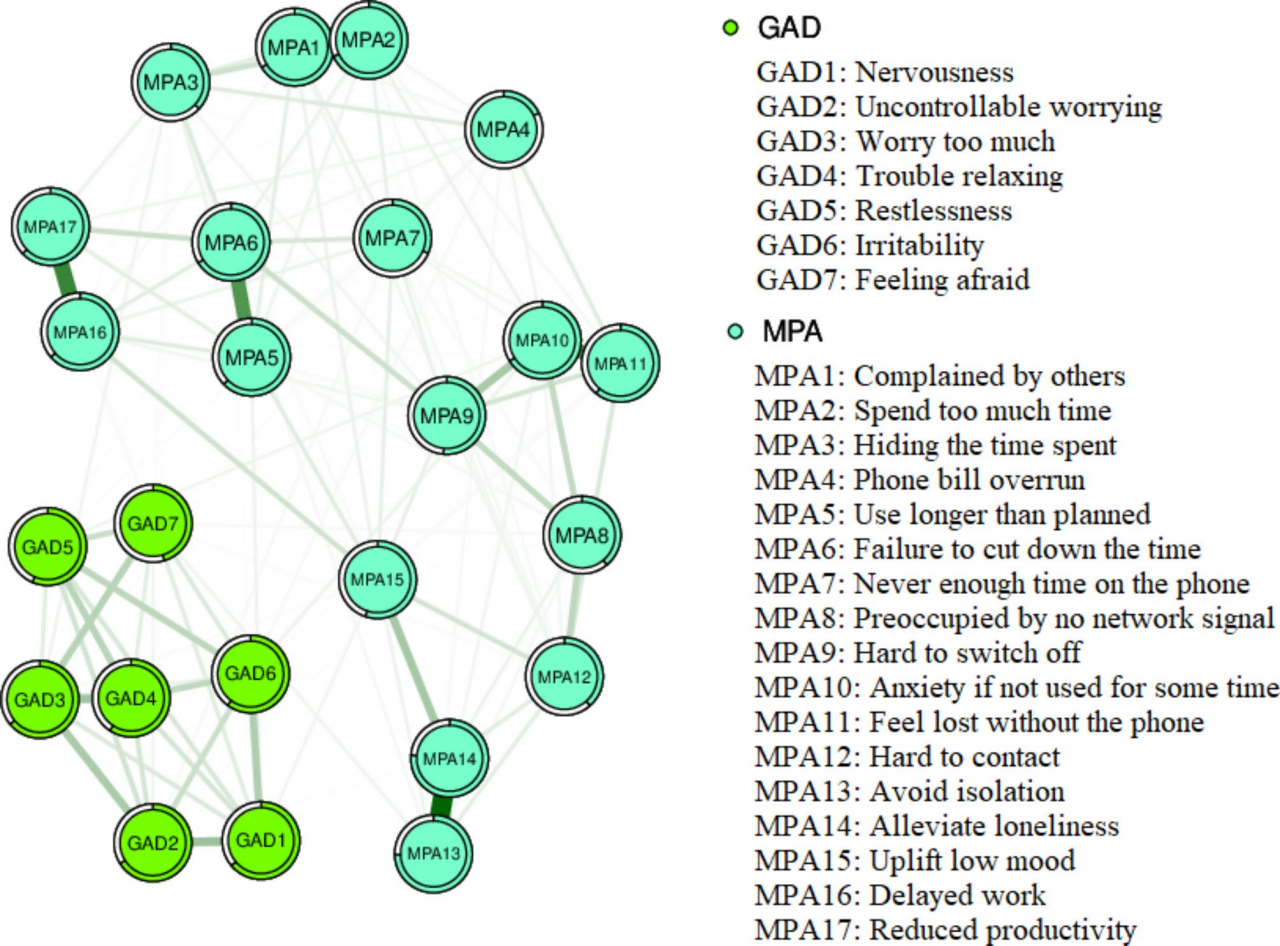
Network of mobile phone addiction and anxiety symptoms in rural Chinese adolescents. Note: Green nodes indicate GAD-7 items and blue nodes indicate MPA items. Green edges indicate positive correlations

The network constituted by mobile phone addiction and anxiety symptoms is shown in Fig. 1. Within the whole network, there is a positive correlation between each symptom of mobile phone addiction and between each symptom of anxiety. And there is also a positive correlation between each symptom of mobile phone addiction and anxiety. In this study, the strongest linkages were all in the mobile phone addiction symptoms. The edges with the most strongly connected were Avoid isolation and Alleviate loneliness (MPA13-MPA14), followed by the connection between Complained by others and Spend too much time (MPA1-MPA2) and Delayed work and Reduced productivity (MPA16-MPA17). The detailed edge weight values are provided in Supplementary Table 2 . The foretelling ability of symptoms is shown in Fig. 1 by a pie chart in the form of a ring. The specific values are provided in Supplementary Table 1 .

**Fig. 2:**
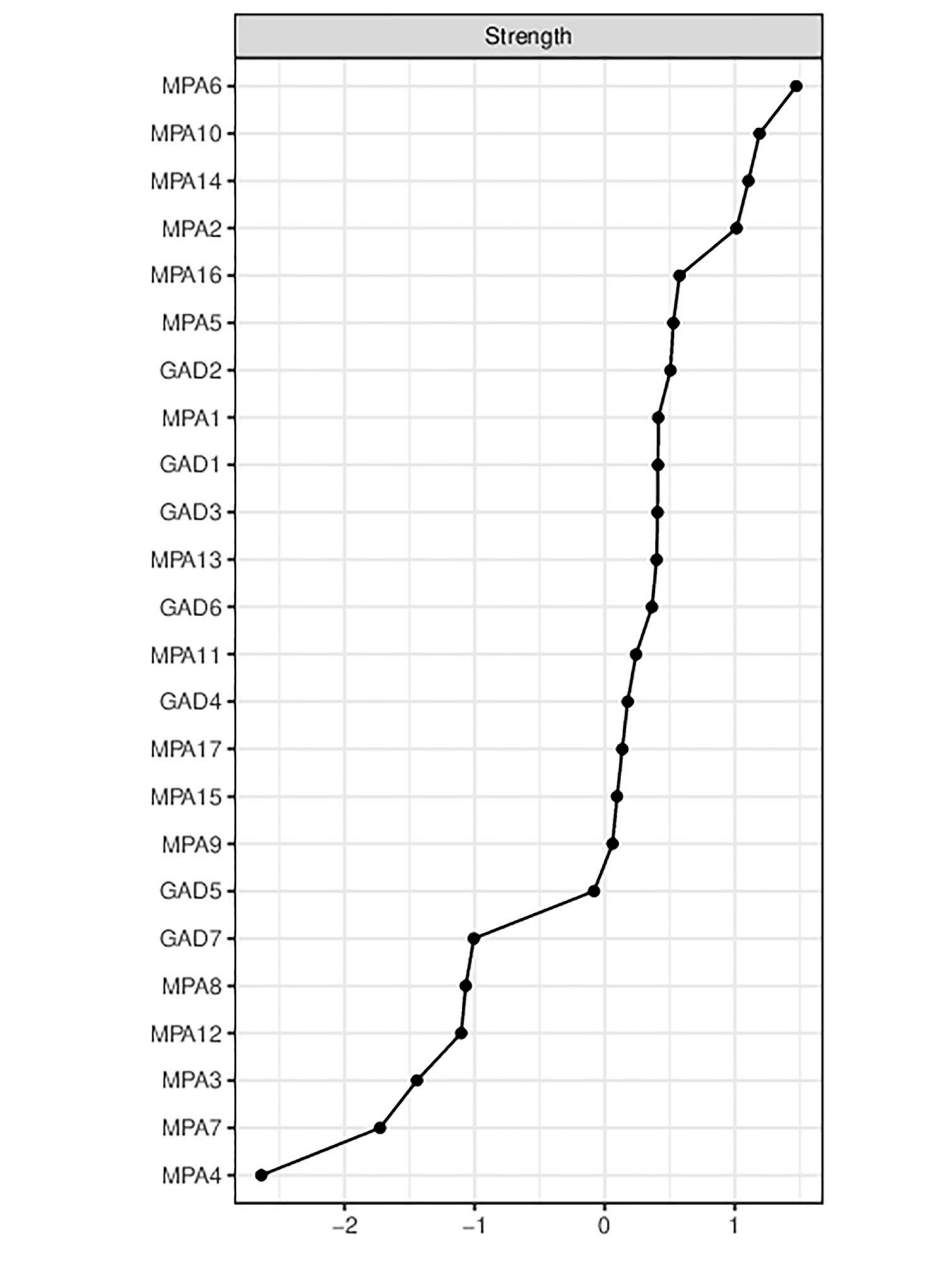
Node strength centrality in the network

**Fig. 3:**
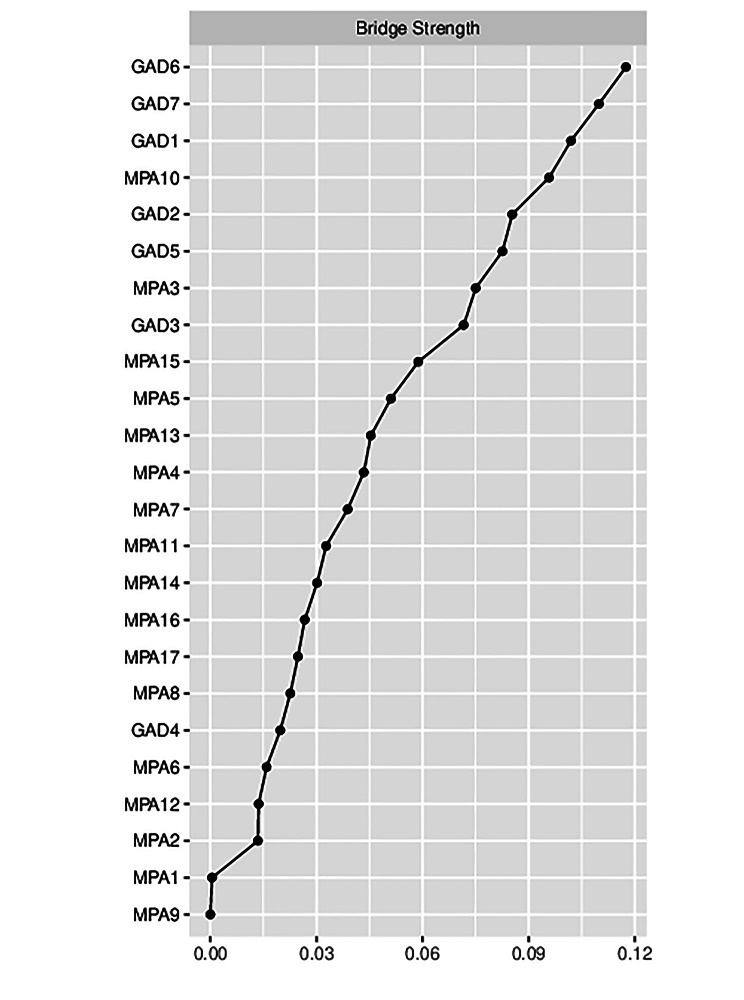
Bridge centrality indices of cell phone addiction and anxiety symptoms in rural Chinese adolescents

In the network of mobile phone addiction and anxiety symptoms, the highest strength symptoms were in the mobile phone addiction symptoms. The highest strength symptom was Failure to cut down the time (MPA6), which was at the core of the network. The next highest symptoms were Anxiety if not used for some time (MPA10), Alleviate loneliness (MPA14), and Hard to contact (MPA2). Among the anxiety symptoms, the highest intensity symptom was Uncontrollable worrying (GAD2). The strength index is shown in Fig. 2. In terms of bridge strength, the symptoms with higher bridge strength mostly belonged to anxiety symptoms. The highest strengths were Irritability (GAD6) and Feeling afraid (GAD7), followed by Nervousness (GAD1) and Anxiety if not used for some time (MPA10) (Fig. 3).

### Network accuracy and stability

**Fig. 4:**
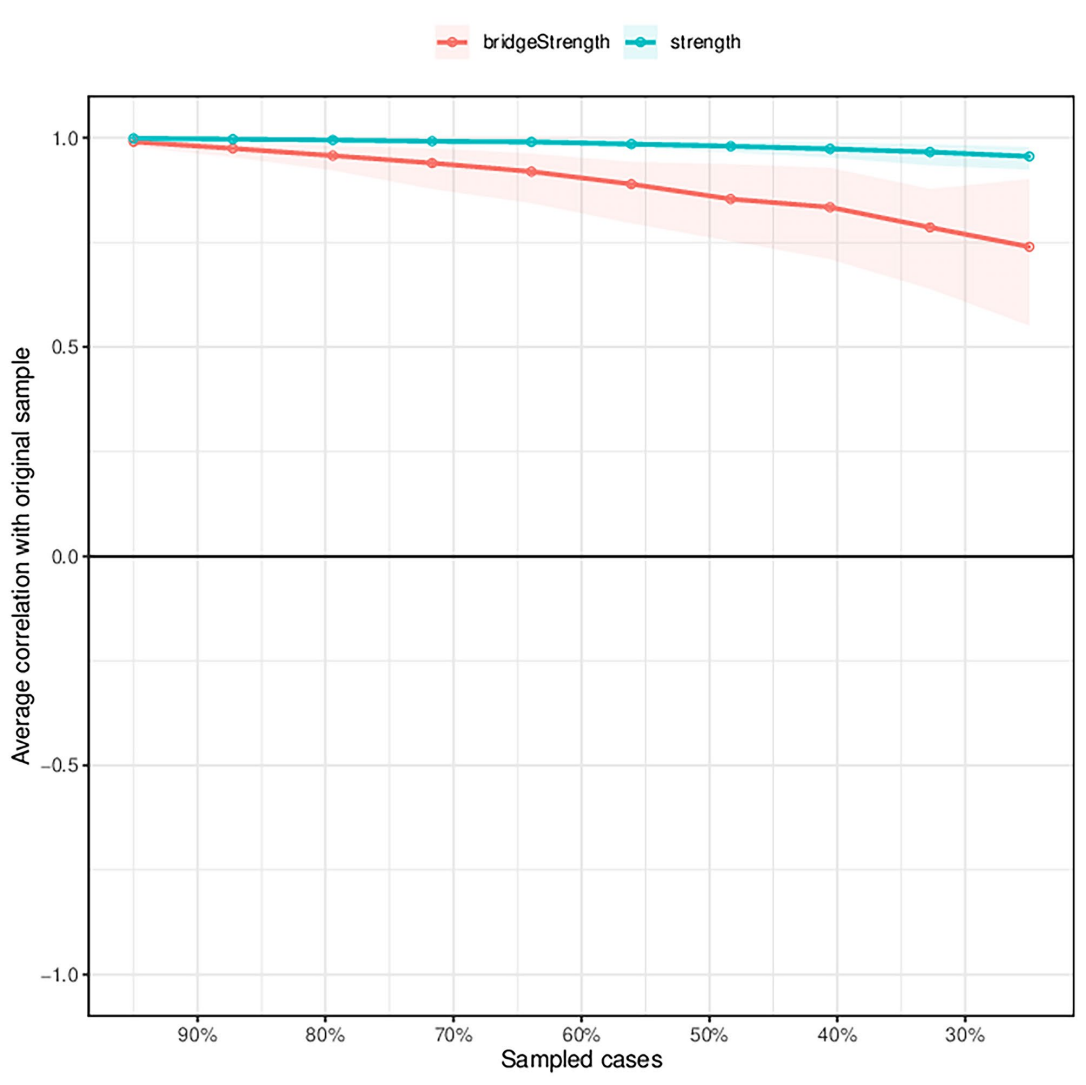
Estimating the stability of the network structure using the case-drop subset bootstrap method. Note: The X-axis represents the percentage of cases using the original sample. Y-axis displays the mean correlation between the original network’s centrality index and the re-estimated network’s index

By testing the stability of the network analysis, we discovered that the stability of the network structure was extremely stable (CS-C = 0.75), indicating that even if 75% of the samples were discarded, the network structure would not change significantly (Fig. 4). As illustrated in Supplementary Fig. 1 , the bootstrap difference test revealed that most comparisons between node strengths were statistically significant.

### Network comparisons

The results of the comparison of network structure among adolescents of different genders demonstrated that gender differences had no significant effect on network models of mobile phone addiction and anxiety symptoms. The distribution of edge weights (*M* = 0.138; *P* = 0.252) and the global strength (*S* = 0.021; *P* = 0.841) of the network did not significantly change across the network models when compared. The figure is shown in Supplementary Figs. 2–3 and Supplementary Tables 3–4 .

### Predicting the value of nodes

**Fig. 5:**
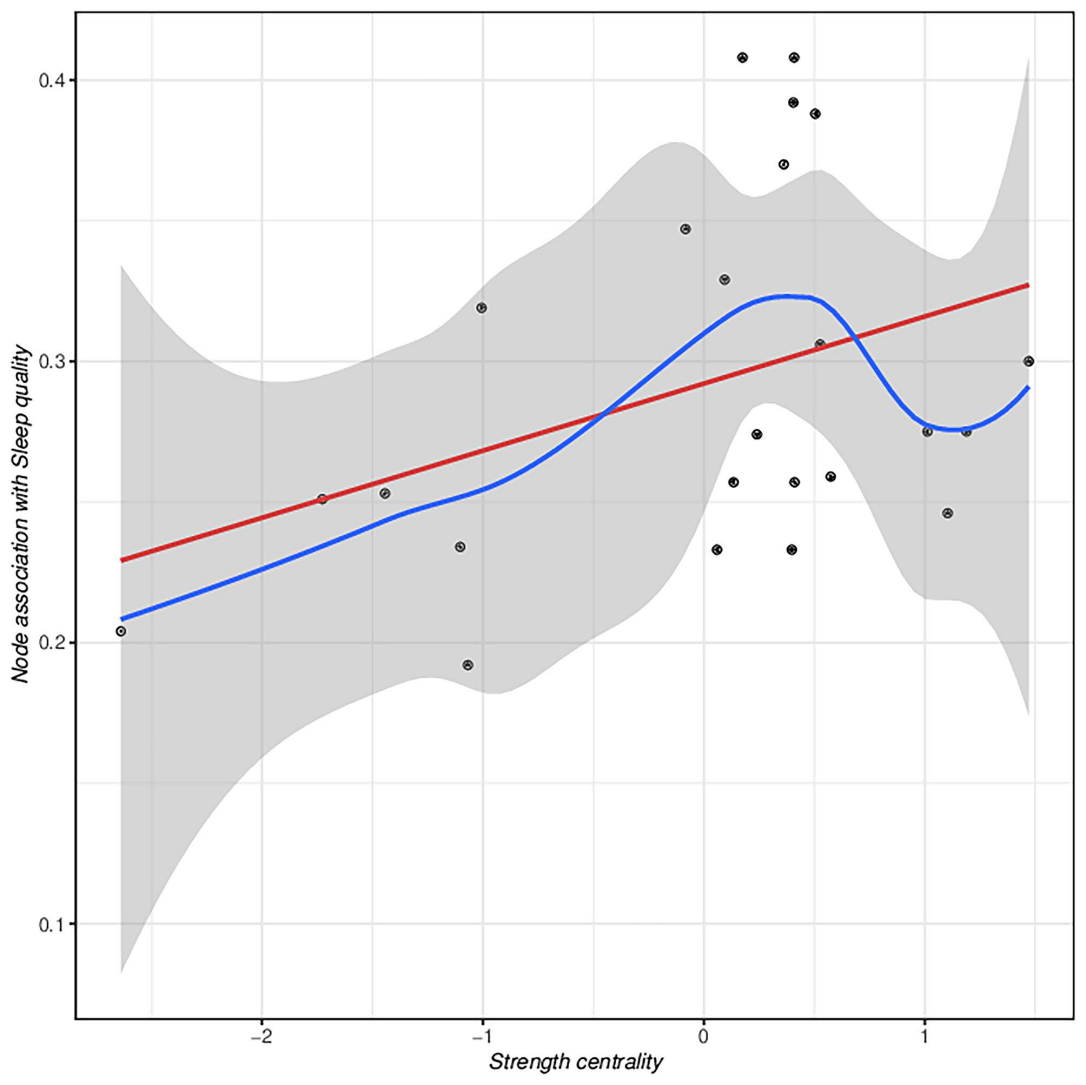
Prediction of sleep quality by central nodes. Note: Each point represents a network node. A point on the x-axis to the right represents a highly central node. A high point on the y-axis represents a node with a high predictive value (strongly related to sleep quality score). The blue line represents the LOWESS estimate result, while the red line represents the linear regression estimation result

First, the correlation between each node in the network and the sleep quality score was calculated and the obtained correlation coefficient was considered as the predictive value of each node for sleep quality. In this study, each node in the network was positively correlated with the sleep quality scores. A high correlation indicates that the node is a high predictor of poor sleep quality. Figure 5 LOWESS curve showing the general trend of positive correlation between predicted values of nodes and strength centrality. The linear regression results, however, indicate that the relationship between the strength centrality of the nodes in the network and the predicted values is not significant (*β* = 0.292, *P* = 0.075). That is, there was no significant correlation between decreased sleep quality and mobile phone addiction and anxiety symptoms among the rural adolescents in this study.

## Discussion

This is a network analysis study on mobile phone addiction and anxiety symptoms among rural Chinese adolescents. In this study, we aimed to examine the relationship between mobile phone addiction and anxiety symptoms through network analysis to determine the central symptoms of this network model and the prediction of highly central nodes on sleep quality. The results of the analysis showed that nodes’ Failure to cut down the time (MPA6), Anxiety if not used for some time (MPA10), and Alleviate loneliness (MPA14) were the most influential symptoms in the mobile phone addiction-anxiety network. The bridge symptoms connecting mobile phone addiction and anxiety symptoms are nodes Irritability (GAD6), Feeling afraid (GAD7), Nervousness (GAD1), and Anxiety if not used for some time (MPA10). In addition, high-strength nodes in the mobile phone addiction-anxiety symptom network were not predictive of sleep quality in rural adolescents.

The most significant symptoms in the mobile phone addiction-anxiety symptom network are concentrated in the nodes of mobile phone addiction, such as node Failure to cut down the time. This may be related to weaker self-control. Numerous studies examined the relationship between self-control and mobile phone addiction, and mobile phone addiction and found that mobile phone addiction has a high correlation with self-control [48–50]. These suggest that the lower the self-control, the greater the likelihood of mobile phone addiction [51, 52]. The more serious the mobile phone addiction is, the more the interpersonal problems of the individual, and then the individual’s anxiety will be induced [53]. Moreover, the features of the phone itself and the Internet access it provides can alleviate anxiety [54], so the more anxious individuals will be unable to resist the temptation of the phone because it is like a pacifier for them, and they need it more for psychological comfort.

Node Anxiety if not used for some time is also one of the central symptoms of the mobile phone addiction-anxiety symptom network, which is consistent with the findings of Elhai et al. [16]. Many studies on the association between problematic mobile phone use and mental health symptoms suggest that high levels of anxiety are positively associated with habitual smartphone-checking behaviors, which may lead to increased addiction to mobile phone use [49, 55]. Social networks are considered a way to meet the demands of relationships [56]. Some research suggests that people who have a strong desire to participate in what others are doing will be prone to a fear of being excluded from social networks [57, 58], and this anxious thought can lead phone users to stay connected to social networks by using their phones frequently to alleviate anxiety and satisfy their need to feel a sense of social belonging [49]. Conversely, anxiety can arise if one leaves the phone behind.

In addition, Alleviate loneliness (i.e. relieving loneliness by chatting with others on mobile phones) is also an influential symptom of the mobile phone addiction-anxiety symptom network, which is consistent with the findings of Yue et al. [59]. Loneliness is a subjective state, usually understood as a negative emotional reaction caused by the difference between a person’s satisfactory interpersonal communication level and the actual interpersonal communication level [60], and a lot studies have proved that loneliness is related to mobile phone addiction. Enez Darcin et al. point out that people who often feel lonely are more likely to rely on the Internet [61]. Other studies have also shown that loneliness significantly predicts mobile phone use patterns and the severity of mobile phone addiction [62]. Through the ‘compensatory Internet use mode’, Kardefelt-Winther puts forward the reason why loneliness leads to mobile phone addiction, that is, people will use mobile phones to escape real-life problems and alleviate negative emotions (including loneliness) [63]. Moreover, lonely people also experience more anxiety [64, 65]. In contrast, most rural adolescents, whose parents work outside the home, live with their grandparents. They are more likely to be neglected and to feel negative emotions such as loneliness [66]. Therefore, they may use mobile phones frequently to relieve their anxiety and to satisfy their psychological need to interact with or be included by others. The Chinese government has also done a lot to address the current situation of rural youths, such as creating a mental health service system for adolescents, but it is still important to continuously focus on the state of rural adolescents’ lives, studies and friendships to alleviate their negative emotions.

In our network, the most influential bridge symptoms were nodes Irritability and Feeling afraid, indicating that those nodes were most closely associated with mobile phone addiction. Some studies have shown that during COVID-19, adolescents become more irritable and may increase their mobile phone use to vent their emotions [67]. And adolescents who feel fearful may be more introverted, anxious or lonely and want to escape from the real world more, so they spend more time on their phones [68–70]. So this would explain why feeling scared would be a bridging symptom between mobile phone addiction and anxiety.

Our study found that mobile phone addiction and anxiety symptom networks were not predictive of sleep, which is not quite in line with previous research findings [71]. The reason for this may be due to the fact that this study investigated mainly middle school students, who are under more academic pressure and students generally have shorter sleep times, and the difference in sleep duration was not significant. However, sleep is particularly important for middle school students’ health and still needs to be given attention.

Our study has some limitations. First, the use of self-report questionnaires to measure mobile phone addiction and anxiety symptoms, rather than by systematic diagnosis, may result in some bias. Second, because the data were collected in a cross-sectional study design, it was not possible to determine causality. Future longitudinal studies are needed to explore the causal relationship between mobile phone addiction and anxiety symptoms over time. Third, we did not investigate issues such as the content of adolescents’ mobile phone use and parental supervision methods, and we failed to exclude the influence of other psychological problems when investigating anxiety symptoms, which should be covered more rigorously in the questionnaire in subsequent studies. Fourth, our data were collected after the onset of the COVID-19 epidemic and were not compared with pre-epidemic data. Therefore, there may be limitations in generalizing to non-epidemic periods.

In conclusion, the most influential symptoms in the network analysis of mobile phone addiction and anxiety symptoms are mainly concentrated in the part of mobile phone addiction. This allows our research to provide some basis for preventive measures that focusing on diverse factors in mobile phone addiction. And now, due to the outbreak of COVID-19, the learning mode of online teaching has increased the time spent using mobile phones, which makes it more necessary for us to monitor the impact of smartphones on the mental health of rural adolescents. Therefore, we may be able to reduce adolescents’ symptoms of mobile phone addiction and anxiety through timely educational monitoring and targeted psychological intervention.

## Electronic supplementary material

Below is the link to the electronic supplementary material.

Supplementary Material 1: Supplemental results

## Data Availability

The datasets used and/or analysed during the current study are available from the corresponding author on reasonable request.

## Abbreviations

MPAI: Mobile Phone Addiction Index scale
GAD-7: The Chinese version of the seven-item Generalized Anxiety Inventory
PSQI: Pittsburgh Sleep Quality Index scale
SD: Standard Deviation
