# Supplementary material for: Network structure of mobile phone addiction and anxiety symptoms among rural Chinese adolescents
Source: BMC Psychiatry. 2023 Jul 10;23:491. doi: 10.1186/s12888-023-04971-x (PMC10332091; doi:10.1186/s12888-023-04971-x)
Supplement: Supplementary file 1 — Supplementary Material 1: Supplemental results [file 12888_2023_4971_MOESM1_ESM.docx]

**Supplementary Information**

**Supplementary figure and table legends**

**Supplementary Table 1: Means, standard deviations, maxima, minima, skewness**

**Supplementary Table 2: Weighted adjacency matrix of Mobile phone addiction symptoms-anxiety symptoms**

**Supplementary Figure 1: Non-parametric bootstrap difference test for node strength**

**Supplementary Figure 2: Network structure of rural Chinese adolescents of different genders**

**Supplementary Table 3: Weighted adjacency matrix of Mobile phone addiction symptoms-anxiety symptoms in male**

**Supplementary Table 4: Weighted adjacency matrix of Mobile phone addiction symptoms-anxiety symptoms in female**

**Supplementary Figure 3: Comparison of network attributes of participants in different gender.**

| **Supplementary Table 1:** **Means, standard deviations, maxima, minima, skewness** | | | | | | | |
| --- | --- | --- | --- | --- | --- | --- | --- |
| variable | M | SD | Min | Max | Skewness | Kurtosis | Predictive value |
| **Mobile phone addiction symptoms** |  |  |  |  |  |  |  |
| Complained by others | 2.64 | 1.243 | 1 | 5 | 0.416 | -0.791 | 0.663 |
| Spend too much time | 2.51 | 1.264 | 1 | 5 | 0.459 | -0.836 | 0.681 |
| Hiding the time spent | 1.88 | 1.172 | 1 | 5 | 1.27 | 0.666 | 0.369 |
| Phone bill overrun | 1.35 | 0.817 | 1 | 5 | 2.771 | 7.824 | 0.180 |
| Use longer than planned | 2.91 | 1.464 | 1 | 5 | 0.088 | -1.395 | 0.628 |
| Failure to cut down the time | 2.68 | 1.457 | 1 | 5 | 0.345 | -1.277 | 0.661 |
| Never enough time on the phone | 2.07 | 1.348 | 1 | 5 | 1.048 | -0.205 | 0.314 |
| Preoccupied by no network signal | 2.19 | 1.4 | 1 | 5 | 0.87 | -0.622 | 0.390 |
| Hard to switch off | 2.11 | 1.423 | 1 | 5 | 0.97 | -0.53 | 0.518 |
| Anxiety if not used for some time | 1.83 | 1.233 | 1 | 5 | 1.403 | 0.808 | 0.662 |
| feel lost without the phone | 1.66 | 1.115 | 1 | 5 | 1.789 | 2.282 | 0.609 |
| Hard to contact | 2.25 | 1.407 | 1 | 5 | 0.754 | -0.818 | 0.392 |
| Avoid isolation | 2.18 | 1.403 | 1 | 5 | 0.878 | -0.603 | 0.747 |
| Alleviate loneliness | 2.37 | 1.437 | 1 | 5 | 0.652 | -0.959 | 0.764 |
| Uplift low mood | 2.81 | 1.438 | 1 | 5 | 0.165 | -1.299 | 0.552 |
| Delayed work | 2.52 | 1.409 | 1 | 5 | 0.503 | -1.046 | 0.637 |
| Reduced productivity | 2.51 | 1.379 | 0 | 5 | 0.498 | -1.003 | 0.628 |
| **Anxiety symptoms** |  |  |  |  |  |  |  |
| Nervousness | 0.73 | 0.843 | 0 | 3 | 1.092 | 0.636 | 0.639 |
| Uncontrollable worrying | 0.62 | 0.856 | 0 | 3 | 1.341 | 1.009 | 0.660 |
| Worry too much | 0.62 | 0.854 | 0 | 3 | 1.337 | 1.015 | 0.640 |
| Trouble relaxing | 0.65 | 0.855 | 0 | 3 | 1.27 | 0.882 | 0.593 |
| Restlessness | 0.52 | 0.777 | 0 | 3 | 1.541 | 1.874 | 0.562 |
| Irritability | 0.63 | 0.85 | 0 | 3 | 1.34 | 1.11 | 0.601 |
| Felling afraid | 0.64 | 0.909 | 0 | 3 | 1.353 | 0.867 | 0.454 |

| **Supplementary Table 2: Weighted adjacency matrix of Mobile phone addiction symptoms-anxiety symptoms** | | | | | | | | | | | | | | | | | | | | | | | | |
| --- | --- | --- | --- | --- | --- | --- | --- | --- | --- | --- | --- | --- | --- | --- | --- | --- | --- | --- | --- | --- | --- | --- | --- | --- |
|  | MPA1 | MPA2 | MPA3 | MAP4 | MPA5 | MPA6 | MPA7 | MPA8 | MPA9 | MPA10 | MPA11 | MPA12 | MPA13 | MPA14 | MPA15 | MPA16 | MPA17 | GAD1 | GAD2 | GAD3 | GAD4 | GAD5 | GAD6 | GAD7 |
| MPA1 | 0.000 | 0.619 | 0.076 | 0.026 | 0.070 | 0.002 | 0.053 | 0.000 | 0.000 | 0.000 | 0.010 | 0.036 | 0.000 | 0.000 | 0.051 | 0.025 | 0.019 | 0.000 | 0.000 | 0.000 | 0.000 | 0.000 | 0.000 | 0.000 |
| MPA2 | 0.619 | 0.000 | 0.136 | 0.033 | 0.043 | 0.050 | 0.061 | 0.000 | 0.025 | 0.049 | 0.026 | 0.024 | 0.000 | 0.000 | 0.006 | 0.000 | 0.007 | 0.000 | 0.000 | 0.000 | 0.000 | 0.013 | 0.000 | 0.000 |
| MPA3 | 0.076 | 0.136 | 0.000 | 0.085 | 0.044 | 0.089 | 0.017 | 0.000 | 0.035 | 0.000 | 0.007 | 0.000 | 0.026 | 0.000 | 0.000 | 0.025 | 0.049 | 0.000 | 0.019 | 0.043 | 0.000 | 0.000 | 0.000 | 0.013 |
| MAP4 | 0.026 | 0.033 | 0.085 | 0.000 | 0.016 | 0.032 | 0.036 | 0.042 | 0.000 | 0.000 | 0.081 | 0.004 | 0.021 | 0.002 | 0.000 | 0.000 | 0.037 | 0.000 | 0.000 | 0.016 | 0.000 | 0.006 | 0.022 | 0.000 |
| MPA5 | 0.070 | 0.043 | 0.044 | 0.016 | 0.000 | 0.483 | 0.018 | 0.028 | 0.018 | 0.000 | 0.000 | 0.000 | 0.000 | 0.000 | 0.081 | 0.084 | 0.073 | 0.045 | 0.006 | 0.000 | 0.000 | 0.000 | 0.000 | 0.000 |
| MPA6 | 0.002 | 0.050 | 0.089 | 0.032 | 0.483 | 0.000 | 0.108 | 0.000 | 0.136 | 0.003 | 0.018 | 0.000 | 0.000 | 0.000 | 0.018 | 0.076 | 0.140 | 0.000 | 0.001 | 0.000 | 0.000 | 0.000 | 0.015 | 0.000 |
| MPA7 | 0.053 | 0.061 | 0.017 | 0.036 | 0.018 | 0.108 | 0.000 | 0.020 | 0.054 | 0.032 | 0.033 | 0.028 | 0.000 | 0.000 | 0.066 | 0.051 | 0.000 | 0.000 | 0.022 | 0.000 | 0.000 | 0.017 | 0.000 | 0.000 |
| MPA8 | 0.000 | 0.000 | 0.000 | 0.042 | 0.028 | 0.000 | 0.020 | 0.000 | 0.165 | 0.165 | 0.000 | 0.178 | 0.029 | 0.050 | 0.015 | 0.000 | 0.018 | 0.000 | 0.000 | 0.000 | 0.000 | 0.000 | 0.000 | 0.023 |
| MPA9 | 0.000 | 0.025 | 0.035 | 0.000 | 0.018 | 0.136 | 0.054 | 0.165 | 0.000 | 0.237 | 0.115 | 0.038 | 0.000 | 0.000 | 0.062 | 0.035 | 0.006 | 0.000 | 0.000 | 0.000 | 0.000 | 0.000 | 0.000 | 0.000 |
| MPA10 | 0.000 | 0.049 | 0.000 | 0.000 | 0.000 | 0.003 | 0.032 | 0.165 | 0.237 | 0.000 | 0.471 | 0.033 | 0.037 | 0.000 | 0.000 | 0.000 | 0.000 | 0.000 | 0.000 | 0.000 | 0.009 | 0.040 | 0.047 | 0.000 |
| MPA11 | 0.010 | 0.026 | 0.007 | 0.081 | 0.000 | 0.018 | 0.033 | 0.000 | 0.115 | 0.471 | 0.000 | 0.100 | 0.002 | 0.000 | 0.038 | 0.016 | 0.008 | 0.000 | 0.014 | 0.000 | 0.000 | 0.005 | 0.014 | 0.000 |
| MPA12 | 0.036 | 0.024 | 0.000 | 0.004 | 0.000 | 0.000 | 0.028 | 0.178 | 0.038 | 0.033 | 0.100 | 0.000 | 0.074 | 0.079 | 0.116 | 0.000 | 0.000 | 0.000 | 0.000 | 0.000 | 0.000 | 0.000 | 0.000 | 0.014 |
| MPA13 | 0.000 | 0.000 | 0.026 | 0.021 | 0.000 | 0.000 | 0.000 | 0.029 | 0.000 | 0.037 | 0.002 | 0.074 | 0.000 | 0.704 | 0.047 | 0.000 | 0.000 | 0.000 | 0.003 | 0.000 | 0.000 | 0.000 | 0.000 | 0.042 |
| MPA14 | 0.000 | 0.000 | 0.000 | 0.002 | 0.000 | 0.000 | 0.000 | 0.050 | 0.000 | 0.000 | 0.000 | 0.079 | 0.704 | 0.000 | 0.243 | 0.000 | 0.000 | 0.000 | 0.020 | 0.000 | 0.010 | 0.000 | 0.000 | 0.000 |
| MPA15 | 0.051 | 0.006 | 0.000 | 0.000 | 0.081 | 0.018 | 0.066 | 0.015 | 0.062 | 0.000 | 0.038 | 0.116 | 0.047 | 0.243 | 0.000 | 0.124 | 0.006 | 0.037 | 0.000 | 0.013 | 0.000 | 0.000 | 0.009 | 0.000 |
| MPA16 | 0.025 | 0.000 | 0.025 | 0.000 | 0.084 | 0.076 | 0.051 | 0.000 | 0.035 | 0.000 | 0.016 | 0.000 | 0.000 | 0.000 | 0.124 | 0.000 | 0.553 | 0.006 | 0.000 | 0.000 | 0.000 | 0.001 | 0.001 | 0.018 |
| MPA17 | 0.019 | 0.007 | 0.049 | 0.037 | 0.073 | 0.140 | 0.000 | 0.018 | 0.006 | 0.000 | 0.008 | 0.000 | 0.000 | 0.000 | 0.006 | 0.553 | 0.000 | 0.014 | 0.000 | 0.000 | 0.000 | 0.000 | 0.010 | 0.000 |
| GAD1 | 0.000 | 0.000 | 0.000 | 0.000 | 0.045 | 0.000 | 0.000 | 0.000 | 0.000 | 0.000 | 0.000 | 0.000 | 0.000 | 0.000 | 0.037 | 0.006 | 0.014 | 0.000 | 0.270 | 0.096 | 0.114 | 0.105 | 0.219 | 0.081 |
| GAD2 | 0.000 | 0.000 | 0.019 | 0.000 | 0.006 | 0.001 | 0.022 | 0.000 | 0.000 | 0.000 | 0.014 | 0.000 | 0.003 | 0.020 | 0.000 | 0.000 | 0.000 | 0.270 | 0.000 | 0.232 | 0.114 | 0.122 | 0.143 | 0.037 |
| GAD3 | 0.000 | 0.000 | 0.043 | 0.016 | 0.000 | 0.000 | 0.000 | 0.000 | 0.000 | 0.000 | 0.000 | 0.000 | 0.000 | 0.000 | 0.013 | 0.000 | 0.000 | 0.096 | 0.232 | 0.000 | 0.260 | 0.076 | 0.055 | 0.196 |
| GAD4 | 0.000 | 0.000 | 0.000 | 0.000 | 0.000 | 0.000 | 0.000 | 0.000 | 0.000 | 0.009 | 0.000 | 0.000 | 0.000 | 0.010 | 0.000 | 0.000 | 0.000 | 0.114 | 0.114 | 0.260 | 0.000 | 0.191 | 0.173 | 0.076 |
| GAD5 | 0.000 | 0.013 | 0.000 | 0.006 | 0.000 | 0.000 | 0.017 | 0.000 | 0.000 | 0.040 | 0.005 | 0.000 | 0.000 | 0.000 | 0.000 | 0.001 | 0.000 | 0.105 | 0.122 | 0.076 | 0.191 | 0.000 | 0.178 | 0.147 |
| GAD6 | 0.000 | 0.000 | 0.000 | 0.022 | 0.000 | 0.015 | 0.000 | 0.000 | 0.000 | 0.047 | 0.014 | 0.000 | 0.000 | 0.000 | 0.009 | 0.001 | 0.010 | 0.219 | 0.143 | 0.055 | 0.173 | 0.178 | 0.000 | 0.094 |
| GAD7 | 0.000 | 0.000 | 0.013 | 0.000 | 0.000 | 0.000 | 0.000 | 0.023 | 0.000 | 0.000 | 0.000 | 0.014 | 0.042 | 0.000 | 0.000 | 0.018 | 0.000 | 0.081 | 0.037 | 0.196 | 0.076 | 0.147 | 0.094 | 0.000 |


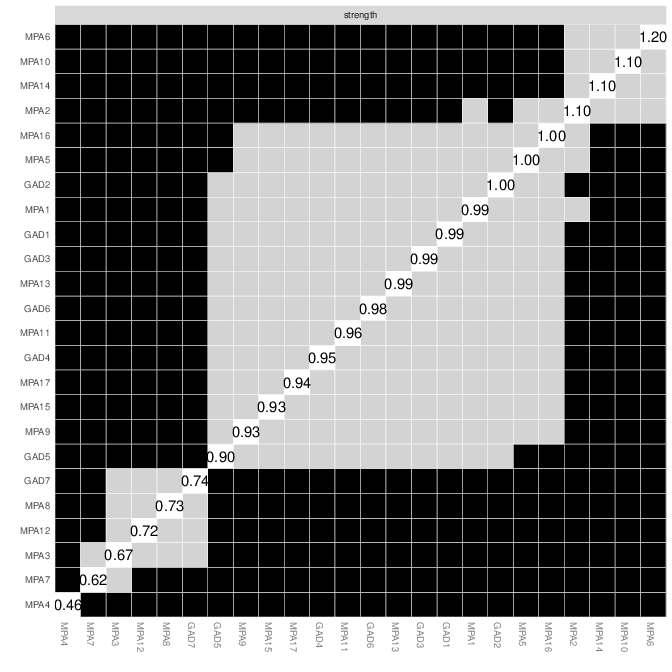


**Supplementary Figure.1 Non-parametric bootstrap difference test for node strength.**

*Note: Gray boxes suggest no differences between nodes, whereas black boxes suggest significant differences (α=0.05). Each node's intensity value is represented by the values provided along the diagonal line.*


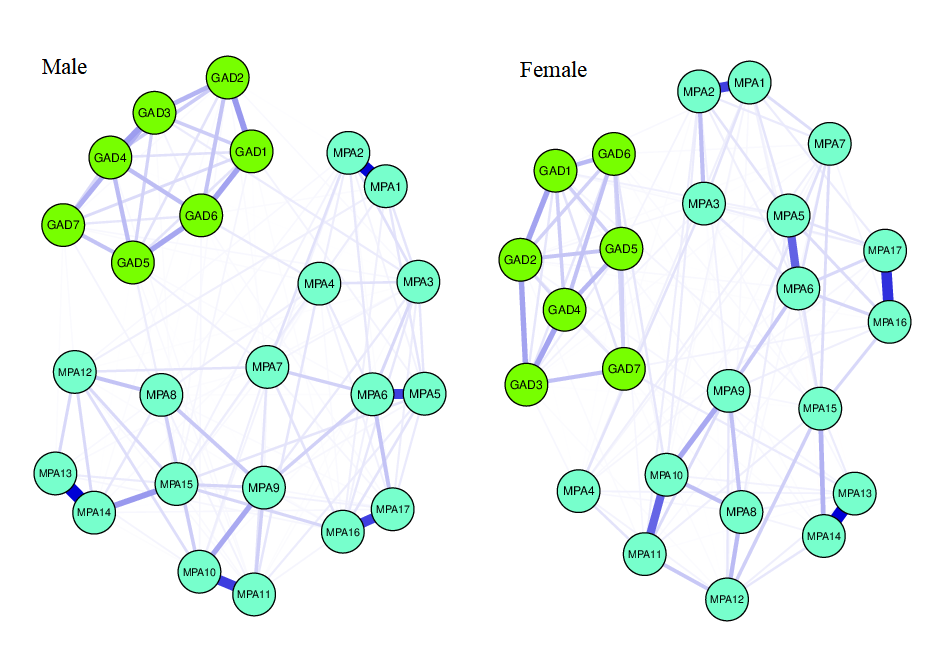


**Supplementary Figure 2: Network structure of rural Chinese adolescents of different genders**

| **Supplementary Table 3: Weighted adjacency matrix of Mobile phone addiction symptoms-anxiety symptoms in male** | | | | | | | | | | | | | | | | | | | | | | | | |
| --- | --- | --- | --- | --- | --- | --- | --- | --- | --- | --- | --- | --- | --- | --- | --- | --- | --- | --- | --- | --- | --- | --- | --- | --- |
|  | MPA1 | MPA2 | MPA3 | MAP4 | MPA5 | MPA6 | MPA7 | MPA8 | MPA9 | MPA10 | MPA11 | MPA12 | MPA13 | MPA14 | MPA15 | MPA16 | MPA17 | GAD1 | GAD2 | GAD3 | GAD4 | GAD5 | GAD6 | GAD7 |
| MPA1 | 0.000 | 0.659 | 0.067 | 0.000 | 0.068 | 0.000 | 0.005 | 0.000 | 0.000 | 0.000 | 0.000 | 0.043 | 0.000 | 0.000 | 0.048 | 0.043 | 0.000 | 0.000 | 0.000 | 0.000 | 0.000 | 0.000 | 0.013 | 0.000 |
| MPA2 | 0.659 | 0.000 | 0.081 | 0.046 | 0.030 | 0.063 | 0.077 | 0.018 | 0.001 | 0.031 | 0.007 | 0.018 | 0.000 | 0.000 | 0.034 | 0.000 | 0.011 | 0.000 | 0.006 | 0.000 | 0.000 | 0.000 | 0.000 | 0.000 |
| MPA3 | 0.067 | 0.081 | 0.000 | 0.078 | 0.069 | 0.103 | 0.028 | 0.000 | 0.058 | 0.000 | 0.009 | 0.009 | 0.015 | 0.000 | 0.000 | 0.062 | 0.047 | 0.000 | 0.016 | 0.051 | 0.001 | 0.000 | 0.000 | 0.021 |
| MAP4 | 0.000 | 0.046 | 0.078 | 0.000 | 0.000 | 0.057 | 0.017 | 0.045 | 0.000 | 0.000 | 0.069 | 0.000 | 0.000 | 0.012 | 0.022 | 0.015 | 0.038 | 0.000 | 0.000 | 0.000 | 0.000 | 0.000 | 0.058 | 0.012 |
| MPA5 | 0.068 | 0.030 | 0.069 | 0.000 | 0.000 | 0.503 | 0.000 | 0.000 | 0.024 | 0.000 | 0.050 | 0.000 | 0.000 | 0.000 | 0.071 | 0.074 | 0.054 | 0.031 | 0.000 | 0.000 | 0.000 | 0.000 | 0.000 | 0.000 |
| MPA6 | 0.000 | 0.063 | 0.103 | 0.057 | 0.503 | 0.000 | 0.120 | 0.008 | 0.120 | 0.010 | 0.010 | 0.000 | 0.000 | 0.000 | 0.000 | 0.036 | 0.156 | 0.000 | 0.000 | 0.000 | 0.000 | 0.000 | 0.000 | 0.014 |
| MPA7 | 0.005 | 0.077 | 0.028 | 0.017 | 0.000 | 0.120 | 0.000 | 0.010 | 0.023 | 0.044 | 0.072 | 0.072 | 0.000 | 0.000 | 0.019 | 0.073 | 0.025 | 0.000 | 0.012 | 0.000 | 0.000 | 0.038 | 0.000 | 0.000 |
| MPA8 | 0.000 | 0.018 | 0.000 | 0.045 | 0.000 | 0.008 | 0.010 | 0.000 | 0.144 | 0.130 | 0.035 | 0.158 | 0.017 | 0.063 | 0.037 | 0.000 | 0.013 | 0.000 | 0.000 | 0.000 | 0.000 | 0.000 | 0.005 | 0.028 |
| MPA9 | 0.000 | 0.001 | 0.058 | 0.000 | 0.024 | 0.120 | 0.023 | 0.144 | 0.000 | 0.226 | 0.125 | 0.001 | 0.000 | 0.002 | 0.110 | 0.020 | 0.022 | 0.005 | 0.000 | 0.000 | 0.000 | 0.004 | 0.000 | 0.000 |
| MPA10 | 0.000 | 0.031 | 0.000 | 0.000 | 0.000 | 0.010 | 0.044 | 0.130 | 0.226 | 0.000 | 0.504 | 0.086 | 0.028 | 0.009 | 0.004 | 0.000 | 0.000 | 0.000 | 0.000 | 0.000 | 0.019 | 0.048 | 0.020 | 0.000 |
| MPA11 | 0.000 | 0.007 | 0.009 | 0.069 | 0.050 | 0.010 | 0.072 | 0.035 | 0.125 | 0.504 | 0.000 | 0.017 | 0.000 | 0.000 | 0.016 | 0.027 | 0.000 | 0.000 | 0.000 | 0.000 | 0.015 | 0.015 | 0.000 | 0.000 |
| MPA12 | 0.043 | 0.018 | 0.009 | 0.000 | 0.000 | 0.000 | 0.072 | 0.158 | 0.001 | 0.086 | 0.017 | 0.000 | 0.103 | 0.101 | 0.100 | 0.010 | 0.000 | 0.000 | 0.000 | 0.000 | 0.000 | 0.000 | 0.000 | 0.013 |
| MPA13 | 0.000 | 0.000 | 0.015 | 0.000 | 0.000 | 0.000 | 0.000 | 0.017 | 0.000 | 0.028 | 0.000 | 0.103 | 0.000 | 0.666 | 0.053 | 0.025 | 0.000 | 0.000 | 0.000 | 0.000 | 0.000 | 0.000 | 0.000 | 0.011 |
| MPA14 | 0.000 | 0.000 | 0.000 | 0.012 | 0.000 | 0.000 | 0.000 | 0.063 | 0.002 | 0.009 | 0.000 | 0.101 | 0.666 | 0.000 | 0.266 | 0.000 | 0.021 | 0.000 | 0.012 | 0.000 | 0.000 | 0.002 | 0.000 | 0.000 |
| MPA15 | 0.048 | 0.034 | 0.000 | 0.022 | 0.071 | 0.000 | 0.019 | 0.037 | 0.110 | 0.004 | 0.016 | 0.100 | 0.053 | 0.266 | 0.000 | 0.106 | 0.036 | 0.027 | 0.016 | 0.010 | 0.006 | 0.000 | 0.000 | 0.000 |
| MPA16 | 0.043 | 0.000 | 0.062 | 0.015 | 0.074 | 0.036 | 0.073 | 0.000 | 0.020 | 0.000 | 0.027 | 0.010 | 0.025 | 0.000 | 0.106 | 0.000 | 0.498 | 0.011 | 0.000 | 0.000 | 0.000 | 0.000 | 0.006 | 0.000 |
| MPA17 | 0.000 | 0.011 | 0.047 | 0.038 | 0.054 | 0.156 | 0.025 | 0.013 | 0.022 | 0.000 | 0.000 | 0.000 | 0.000 | 0.021 | 0.036 | 0.498 | 0.000 | 0.000 | 0.000 | 0.000 | 0.000 | 0.000 | 0.015 | 0.000 |
| GAD1 | 0.000 | 0.000 | 0.000 | 0.000 | 0.031 | 0.000 | 0.000 | 0.000 | 0.005 | 0.000 | 0.000 | 0.000 | 0.000 | 0.000 | 0.027 | 0.011 | 0.000 | 0.000 | 0.269 | 0.131 | 0.078 | 0.074 | 0.242 | 0.094 |
| GAD2 | 0.000 | 0.006 | 0.016 | 0.000 | 0.000 | 0.000 | 0.012 | 0.000 | 0.000 | 0.000 | 0.000 | 0.000 | 0.000 | 0.012 | 0.016 | 0.000 | 0.000 | 0.269 | 0.000 | 0.196 | 0.133 | 0.072 | 0.158 | 0.013 |
| GAD3 | 0.000 | 0.000 | 0.051 | 0.000 | 0.000 | 0.000 | 0.000 | 0.000 | 0.000 | 0.000 | 0.000 | 0.000 | 0.000 | 0.000 | 0.010 | 0.000 | 0.000 | 0.131 | 0.196 | 0.000 | 0.256 | 0.114 | 0.032 | 0.210 |
| GAD4 | 0.000 | 0.000 | 0.001 | 0.000 | 0.000 | 0.000 | 0.000 | 0.000 | 0.000 | 0.019 | 0.015 | 0.000 | 0.000 | 0.000 | 0.006 | 0.000 | 0.000 | 0.078 | 0.133 | 0.256 | 0.000 | 0.175 | 0.172 | 0.100 |
| GAD5 | 0.000 | 0.000 | 0.000 | 0.000 | 0.000 | 0.000 | 0.038 | 0.000 | 0.004 | 0.048 | 0.015 | 0.000 | 0.000 | 0.002 | 0.000 | 0.000 | 0.000 | 0.074 | 0.072 | 0.114 | 0.175 | 0.000 | 0.231 | 0.158 |
| GAD6 | 0.013 | 0.000 | 0.000 | 0.058 | 0.000 | 0.000 | 0.000 | 0.005 | 0.000 | 0.020 | 0.000 | 0.000 | 0.000 | 0.000 | 0.000 | 0.006 | 0.015 | 0.242 | 0.158 | 0.032 | 0.172 | 0.231 | 0.000 | 0.066 |
| GAD7 | 0.000 | 0.000 | 0.021 | 0.012 | 0.000 | 0.014 | 0.000 | 0.028 | 0.000 | 0.000 | 0.000 | 0.013 | 0.011 | 0.000 | 0.000 | 0.000 | 0.000 | 0.094 | 0.013 | 0.210 | 0.100 | 0.158 | 0.066 | 0.000 |

| **Supplementary Table 4: Weighted adjacency matrix of Mobile phone addiction symptoms-anxiety symptoms in female** | | | | | | | | | | | | | | | | | | | | | | | | |
| --- | --- | --- | --- | --- | --- | --- | --- | --- | --- | --- | --- | --- | --- | --- | --- | --- | --- | --- | --- | --- | --- | --- | --- | --- |
|  | MPA1 | MPA2 | MPA3 | MAP4 | MPA5 | MPA6 | MPA7 | MPA8 | MPA9 | MPA10 | MPA11 | MPA12 | MPA13 | MPA14 | MPA15 | MPA16 | MPA17 | GAD1 | GAD2 | GAD3 | GAD4 | GAD5 | GAD6 | GAD7 |
| MPA1 | 0.000 | 0.536 | 0.079 | 0.043 | 0.072 | 0.028 | 0.094 | 0.000 | 0.000 | 0.010 | 0.032 | 0.020 | 0.000 | 0.000 | 0.040 | 0.001 | 0.053 | 0.018 | 0.000 | 0.000 | 0.000 | 0.000 | 0.000 | 0.000 |
| MPA2 | 0.536 | 0.000 | 0.182 | 0.011 | 0.077 | 0.045 | 0.059 | 0.000 | 0.042 | 0.055 | 0.049 | 0.005 | 0.000 | 0.000 | 0.000 | 0.000 | 0.000 | 0.000 | 0.000 | 0.000 | 0.000 | 0.029 | 0.000 | 0.000 |
| MPA3 | 0.079 | 0.182 | 0.000 | 0.077 | 0.031 | 0.084 | 0.005 | 0.004 | 0.008 | 0.000 | 0.000 | 0.000 | 0.022 | 0.000 | 0.003 | 0.001 | 0.053 | 0.007 | 0.026 | 0.033 | 0.000 | 0.000 | 0.000 | 0.000 |
| MAP4 | 0.043 | 0.011 | 0.077 | 0.000 | 0.028 | 0.017 | 0.046 | 0.022 | 0.000 | 0.000 | 0.078 | 0.034 | 0.035 | 0.000 | 0.000 | 0.000 | 0.026 | 0.000 | 0.003 | 0.022 | 0.000 | 0.004 | 0.000 | 0.000 |
| MPA5 | 0.072 | 0.077 | 0.031 | 0.028 | 0.000 | 0.437 | 0.025 | 0.054 | 0.004 | 0.000 | 0.000 | 0.000 | 0.000 | 0.000 | 0.078 | 0.098 | 0.080 | 0.042 | 0.000 | 0.003 | 0.000 | 0.000 | 0.000 | 0.000 |
| MPA6 | 0.028 | 0.045 | 0.084 | 0.017 | 0.437 | 0.000 | 0.089 | 0.000 | 0.155 | 0.000 | 0.000 | 0.000 | -0.002 | -0.005 | 0.049 | 0.116 | 0.109 | 0.000 | 0.019 | 0.000 | 0.000 | 0.000 | 0.006 | 0.000 |
| MPA7 | 0.094 | 0.059 | 0.005 | 0.046 | 0.025 | 0.089 | 0.000 | 0.021 | 0.087 | 0.014 | 0.000 | 0.000 | 0.000 | 0.000 | 0.098 | 0.025 | 0.000 | 0.000 | 0.010 | 0.013 | 0.000 | 0.000 | 0.016 | 0.000 |
| MPA8 | 0.000 | 0.000 | 0.004 | 0.022 | 0.054 | 0.000 | 0.021 | 0.000 | 0.173 | 0.179 | 0.000 | 0.189 | 0.046 | 0.032 | 0.000 | 0.000 | 0.001 | 0.000 | 0.000 | 0.000 | 0.000 | 0.000 | 0.000 | 0.011 |
| MPA9 | 0.000 | 0.042 | 0.008 | 0.000 | 0.004 | 0.155 | 0.087 | 0.173 | 0.000 | 0.244 | 0.102 | 0.070 | 0.000 | 0.000 | 0.024 | 0.046 | 0.000 | 0.000 | 0.000 | 0.000 | 0.000 | 0.000 | 0.000 | 0.000 |
| MPA10 | 0.010 | 0.055 | 0.000 | 0.000 | 0.000 | 0.000 | 0.014 | 0.179 | 0.244 | 0.000 | 0.429 | 0.000 | 0.035 | 0.000 | 0.000 | 0.000 | 0.000 | 0.000 | 0.000 | 0.000 | 0.000 | 0.023 | 0.070 | 0.000 |
| MPA11 | 0.032 | 0.049 | 0.000 | 0.078 | 0.000 | 0.000 | 0.000 | 0.000 | 0.102 | 0.429 | 0.000 | 0.155 | 0.008 | 0.000 | 0.048 | 0.000 | 0.019 | 0.000 | 0.015 | 0.000 | 0.000 | 0.000 | 0.024 | 0.000 |
| MPA12 | 0.020 | 0.005 | 0.000 | 0.034 | 0.000 | 0.000 | 0.000 | 0.189 | 0.070 | 0.000 | 0.155 | 0.000 | 0.044 | 0.067 | 0.135 | 0.000 | 0.000 | 0.000 | 0.000 | 0.012 | 0.000 | 0.000 | 0.002 | 0.011 |
| MPA13 | 0.000 | 0.000 | 0.022 | 0.035 | 0.000 | -0.002 | 0.000 | 0.046 | 0.000 | 0.035 | 0.008 | 0.044 | 0.000 | 0.712 | 0.053 | 0.000 | 0.000 | 0.000 | 0.025 | 0.000 | 0.000 | 0.000 | 0.000 | 0.058 |
| MPA14 | 0.000 | 0.000 | 0.000 | 0.000 | 0.000 | -0.005 | 0.000 | 0.032 | 0.000 | 0.000 | 0.000 | 0.067 | 0.712 | 0.000 | 0.214 | 0.000 | 0.000 | 0.019 | 0.000 | 0.000 | 0.011 | 0.000 | 0.010 | 0.000 |
| MPA15 | 0.040 | 0.000 | 0.003 | 0.000 | 0.078 | 0.049 | 0.098 | 0.000 | 0.024 | 0.000 | 0.048 | 0.135 | 0.053 | 0.214 | 0.000 | 0.108 | 0.000 | 0.030 | 0.000 | 0.005 | 0.000 | 0.000 | 0.015 | 0.000 |
| MPA16 | 0.001 | 0.000 | 0.001 | 0.000 | 0.098 | 0.116 | 0.025 | 0.000 | 0.046 | 0.000 | 0.000 | 0.000 | 0.000 | 0.000 | 0.108 | 0.000 | 0.580 | 0.000 | 0.009 | 0.000 | 0.000 | 0.002 | 0.000 | 0.033 |
| MPA17 | 0.053 | 0.000 | 0.053 | 0.026 | 0.080 | 0.109 | 0.000 | 0.001 | 0.000 | 0.000 | 0.019 | 0.000 | 0.000 | 0.000 | 0.000 | 0.580 | 0.000 | 0.019 | 0.000 | 0.000 | 0.000 | 0.000 | 0.000 | 0.000 |
| GAD1 | 0.018 | 0.000 | 0.007 | 0.000 | 0.042 | 0.000 | 0.000 | 0.000 | 0.000 | 0.000 | 0.000 | 0.000 | 0.000 | 0.019 | 0.030 | 0.000 | 0.019 | 0.000 | 0.258 | 0.071 | 0.138 | 0.134 | 0.194 | 0.078 |
| GAD2 | 0.000 | 0.000 | 0.026 | 0.003 | 0.000 | 0.019 | 0.010 | 0.000 | 0.000 | 0.000 | 0.015 | 0.000 | 0.025 | 0.000 | 0.000 | 0.009 | 0.000 | 0.258 | 0.000 | 0.256 | 0.100 | 0.163 | 0.128 | 0.062 |
| GAD3 | 0.000 | 0.000 | 0.033 | 0.022 | 0.003 | 0.000 | 0.013 | 0.000 | 0.000 | 0.000 | 0.000 | 0.012 | 0.000 | 0.000 | 0.005 | 0.000 | 0.000 | 0.071 | 0.256 | 0.000 | 0.255 | 0.050 | 0.072 | 0.177 |
| GAD4 | 0.000 | 0.000 | 0.000 | 0.000 | 0.000 | 0.000 | 0.000 | 0.000 | 0.000 | 0.000 | 0.000 | 0.000 | 0.000 | 0.011 | 0.000 | 0.000 | 0.000 | 0.138 | 0.100 | 0.255 | 0.000 | 0.198 | 0.172 | 0.060 |
| GAD5 | 0.000 | 0.029 | 0.000 | 0.004 | 0.000 | 0.000 | 0.000 | 0.000 | 0.000 | 0.023 | 0.000 | 0.000 | 0.000 | 0.000 | 0.000 | 0.002 | 0.000 | 0.134 | 0.163 | 0.050 | 0.198 | 0.000 | 0.143 | 0.127 |
| GAD6 | 0.000 | 0.000 | 0.000 | 0.000 | 0.000 | 0.006 | 0.016 | 0.000 | 0.000 | 0.070 | 0.024 | 0.002 | 0.000 | 0.010 | 0.015 | 0.000 | 0.000 | 0.194 | 0.128 | 0.072 | 0.172 | 0.143 | 0.000 | 0.115 |
| GAD7 | 0.000 | 0.000 | 0.000 | 0.000 | 0.000 | 0.000 | 0.000 | 0.011 | 0.000 | 0.000 | 0.000 | 0.011 | 0.058 | 0.000 | 0.000 | 0.033 | 0.000 | 0.078 | 0.062 | 0.177 | 0.060 | 0.127 | 0.115 | 0.000 |


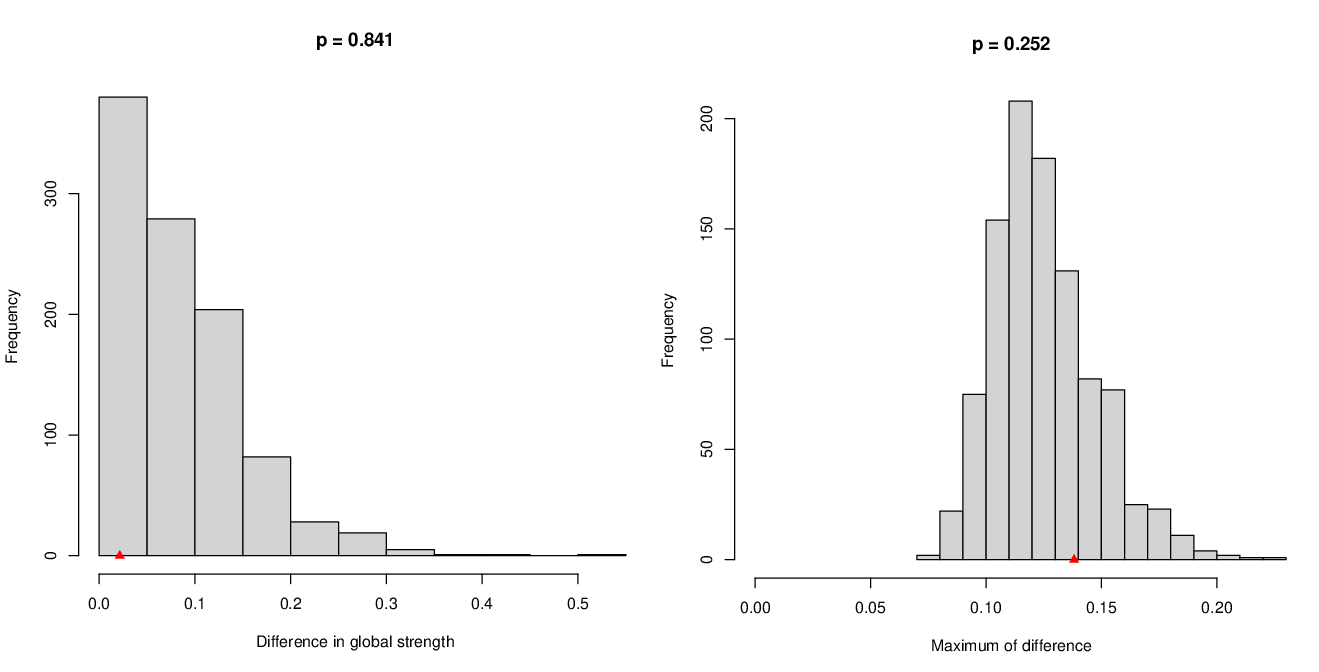


**Supplementary Figure 3: Comparison of network attributes of participants in different gender.**

Left Panel: Plot of bootstrap value of the difference in network global strength, with significant difference (network strength among male participants: 10.992; among female participants: 10.970; S=0.021, *P*=0.841).

Right Panel: Plot of bootstrap value of the maximum difference in any of the edge weights (1000 permutations), differences were statistically significant (M=0.138, *P*=0.252).
